# Urological Malformations Identify the High-Burden Phenotype Among Children Hospitalized for Presumed Urinary Tract Infection: A Retrospective Cohort

**DOI:** 10.3390/diagnostics16132109

**Published:** 2026-07-06

**Authors:** Ana C. Espíritu-Mojarro, Gustavo A. Hernández-Fuentes, Gabriela E. Pedroza-Orozco, José Guzmán-Esquivel, Jesús Venegas-Ramírez, Ileana Y. Ceja-Claro, Daniel A. Montes-Galindo, Carmen A. Sánchez-Ramírez, Mercedes Fuentes-Murguia, Fabian Rojas-Larios, Karmina Sánchez-Meza, Gabriel Ceja-Espíritu, Mario Del-Toro-Equihua, Iván Delgado-Enciso

**Affiliations:** 1 Department of Pediatrics, Mexican Institute of Social Security (IMSS), General Hospital of Zone No. 1, Villa de Alvarez 28984, Mexico; dra.citlalli.pediatra@gmail.com (A.C.E.-M.); dra.gabypedroza@gmail.com (G.E.P.-O.); yunuencejacl@gmail.com (I.Y.C.-C.); 2 Department of Molecular Medicine, School of Medicine, University of Colima, Colima 28040, Mexico; ghfuentes@ucol.mx (G.A.H.-F.); carmen_sanchez@ucol.mx (C.A.S.-R.); fuentes_murguia@ucol.mx (M.F.-M.); frojas@ucol.mx (F.R.-L.); ksmeza@ucol.mx (K.S.-M.); gcejae11@ucol.mx (G.C.-E.); mequihua@ucol.mx (M.D.-T.-E.); 3 State Cancerology Institute of Colima, Health Services of the Mexican Social Security Institute for Welfare (IMSS-BIENESTAR), Colima 28085, Mexico; 4 Faculty of Chemical Sciences, University of Colima, Coquimatlan 28400, Mexico; daniel_montes@ucol.mx; 5 Clinical Epidemiology Research Unit, Mexican Institute of Social Security, Villa de Alvarez 28984, Mexico; jose.esquivel@imss.gob.mx; 6 Department of Nephrology, Instituto Mexicano del Seguro Social (IMSS), Hospital de Zona No. 1, Villa de Álvarez 28984, Mexico; jesus.venegas@imss.gob.mx; 7 Robert Stempel College of Public Health and Social Work, Florida International University, Miami, FL 33199, USA

**Keywords:** pediatric urinary tract infection, renal ultrasound, nephrourological malformations, hospitalization, prognostic stratification, pediatric nephrology

## Abstract

**Background/Objectives:** The clinical utility of renal ultrasound after pediatric urinary tract infection (UTI) remains controversial, particularly because not all ultrasonographic abnormalities have the same prognostic significance. This study aimed to determine whether nephrourological malformations identify the phenotype associated with greater subsequent clinical burden among children hospitalized with presumed UTI and interpretable renal ultrasound findings, and to differentiate this phenotype from non-malformative ultrasound abnormalities. **Methods:** A retrospective single-center hospital-based cohort study was conducted in children aged 2 months to 17 years hospitalized with a clinical diagnosis of UTI at a general hospital of the Mexican Social Security Institute between 2020 and 2025. The cohort included both microbiologically confirmed UTI cases and probable clinical/microbiologically unconfirmed UTI cases. Of 182 registered patients, 130 with interpretable renal ultrasound were included. The primary exposure was the presence of adjudicated nephrourological malformation. As a secondary exposure, within the subgroup without malformation, abnormal non-malformative ultrasound findings were compared with normal ultrasound findings. Outcomes included outpatient follow-up, subspecialty referral, and hospital readmission. Crude associations were expressed as relative risks (RR), and adjusted analyses were estimated using modified Poisson regression with HC3 robust errors. **Results:** Twenty-nine of 130 patients (22.31%) were classified as having nephrourological malformations, and 31 (23.85%) had abnormal non-malformative ultrasound findings. Malformations were associated with higher outpatient follow-up (79.31% vs. 44.55%; RR 1.78, 95% CI 1.34–2.37), greater subspecialty referral (79.31% vs. 49.50%; RR 1.60, 95% CI 1.22–2.10), and increased readmission (44.83% vs. 13.86%; RR 3.23, 95% CI 1.72–6.08). In adjusted models, malformations remained associated with follow-up (aRR 1.72, 95% CI 1.25–2.37), referral (aRR 1.59, 95% CI 1.17–2.16), and readmission (aRR 3.38, 95% CI 1.58–7.23). In contrast, abnormal non-malformative ultrasound findings showed no significant adjusted associations. Microbiologically confirmed UTI was present in 47/130 patients (36.15%), and malformations were more frequent in this subgroup than in probable/non-confirmed clinical UTI (34.04% vs. 15.66%; *p* = 0.027). **Conclusions:** In this single-center hospital-based cohort, subsequent clinical burden was concentrated in the nephrourological malformation phenotype rather than in the broader category of “abnormal ultrasound”. These findings suggest that renal ultrasound may serve as a useful prognostic stratification tool beyond its role as a nonspecific detector of abnormalities following pediatric UTI. Given the observational design, these associations should be confirmed in larger prospective studies.

## 1. Introduction

Urinary tract infection (UTI) is one of the most frequent bacterial infections in pediatrics and a major cause of outpatient visits, emergency care, and hospitalization. Its clinical relevance depends not only on the acute infectious episode itself, but also on the possibility of bacteremia, renal parenchymal involvement, abscess formation, renal scarring, and recurrence [1]. Therefore, UTI should not be interpreted solely as an isolated infectious event, but also as a potential manifestation of underlying structural vulnerability of the urinary tract, with implications for recurrence, renal damage, and complexity of long-term clinical follow-up [1,2].

The diagnostic confirmation of pediatric UTI relies on the convergence of three elements: a compatible clinical presentation, evidence of urinary inflammation on urinalysis, and significant bacteriuria in an appropriately obtained urine culture. Contemporary clinical guidelines continue to consider urine culture the microbiological gold standard, and recent ESPID recommendations for complicated UTI in children maintain that the definition of UTI requires clinical findings, pyuria, and significant bacteriuria [3,4,5]. However, in real-world hospital practice, not all patients have an available culture with recoverable bacterial quantification. The principal microorganism responsible for urinary tract infections in children is *Escherichia coli* [1,2]. In routine clinical practice, it is common for patients to receive treatment for probable UTI despite negative or inconclusive cultures when the clinical presentation is compatible and urinalysis demonstrates leukocyte esterase greater than trace on dipstick and/or more than 5 leukocytes per high-power field on microscopy [6,7,8,9].

Renal imaging also occupies a controversial role in the management of pediatric UTI. The meta-analysis by Yang and colleagues [10] showed that approximately one in every four to five children with a first febrile UTI presents some abnormality on renal ultrasound; however, only a minority exhibit findings clearly relevant for clinical decision-making. At the same time, current guidelines for complicated UTI emphasize that the utility of imaging does not lie in indiscriminately cataloging abnormalities, but rather in identifying children with significant anatomical or functional anomalies, increased risk of renal damage, recurrence, or need for specialized referral. Therefore, the true clinical question is not whether an ultrasound is “abnormal” in descriptive terms, but whether the finding defines a phenotype with prognostic and healthcare implications [11,12,13,14].

Nephrourological malformations and, more broadly, congenital anomalies of the kidney and urinary tract (CAKUT), are particularly important within this framework. These entities represent one of the leading causes of chronic kidney disease and kidney failure in childhood and may result in urinary stasis, obstruction, vesicoureteral reflux, recurrent infections, and the need for longitudinal surveillance by pediatric nephrology or urology services. From both pathophysiological and healthcare organizational perspectives, it is reasonable to expect that children with a malformative phenotype accumulate greater follow-up, more subspecialty referrals, and more hospital readmissions than those with nonspecific or transient ultrasonographic abnormalities; nevertheless, the available literature remains limited [15,16].

The present study adopted an approach aligned with real-world hospital practice. Instead of grouping all abnormal ultrasound findings into a methodologically heterogeneous category, the study explicitly separates nephrourological malformations from non-malformative ultrasonographic abnormalities. Within this context, the following hypothesis was proposed: the greatest subsequent clinical burden is not concentrated in the broad category of “abnormal ultrasound,” but specifically in the structural malformative phenotype. The primary exposure was the presence of nephrourological malformation, whereas the primary outcomes were outpatient follow-up, referral to subspecialty care, and hospital readmission. Accordingly, the primary objective was to determine whether nephrourological malformations identify the phenotype associated with a more complex subsequent clinical trajectory and to differentiate this phenotype from non-malformative ultrasonographic abnormalities. The secondary objective was to evaluate whether this pattern persisted when analyses were restricted to patients with microbiologically confirmed UTI. To reflect real-world pediatric practice, the study was conducted in a single-center hospital setting and included both microbiologically confirmed and probable clinical/microbiologically unconfirmed UTI cases.

## 2. Materials and Methods

### 2.1. Study Design and Setting

A retrospective single-center hospital-based observational cohort study was conducted based on the review of medical records of children hospitalized with a diagnosis of UTI at the General Hospital Zone No. 1 of the Mexican Social Security Institute (IMSS-Colima), during the period from January 2020 to December 2025. The episode for which the patient was admitted and included in the database was defined as the index episode. The study was designed as a real-world hospital-based cohort; therefore, the primary interest was not to validate a diagnostic algorithm, but rather to identify whether the presence of nephrourological malformation was associated with a subsequent clinical trajectory characterized by greater healthcare burden [17].

### 2.2. Study Population and Eligibility Criteria

Patients aged 2 months to 17 years hospitalized with a clinical diagnosis of UTI and with an interpretable renal ultrasound corresponding to the index episode or its related in-hospital evaluation were included. Records without ultrasound, with non-interpretable ultrasound, or with insufficient ultrasonographic information to classify the study as normal or abnormal were excluded.

### 2.3. Operational Definition of Diagnostic Certainty for UTI

In order to reflect real-world clinical practice without sacrificing conceptual rigor, diagnostic certainty for UTI was stratified into two levels. Microbiologically confirmed UTI was defined by the combination of (1) a compatible clinical presentation managed as UTI by the treating team; (2) evidence of urinary inflammation, defined as leukocyte esterase greater than trace on dipstick and/or more than 5 leukocytes per high-power field on microscopy, when such data were available; and (3) a urine culture reported as positive for a recognized uropathogen by the institutional laboratory.

The category of probable clinical UTI or microbiologically unconfirmed UTI included hospitalized patients treated as UTI during the index episode who did not meet formal microbiological confirmation criteria, whether because of negative, unavailable, missing, or inconclusive cultures, but who maintained a compatible clinical presentation and, when available, evidence of urinary inflammation on urinalysis. This methodological decision was adopted because the objective of the study was prognostic rather than diagnostic accuracy; consequently, microbiological certainty was treated as a stratification variable in the analyses rather than as an exclusion criterion for the main cohort.

### 2.4. Ultrasonographic Classification and Adjudication of Malformation

Renal ultrasound was initially classified as normal or abnormal according to the interpretation documented in the medical record. Subsequently, abnormal studies were reviewed to distinguish between malformative structural abnormalities and non-malformative alterations [18,19,20]. Structural patterns compatible with persistent ectasia or pyelectasis, hydronephrosis, ureteral dilation, pyeloureteral dilatation subsequently confirmed as vesicoureteral reflux, ureterocele, posterior urethral valves, complex anomalies, or related anatomical combinations were classified as malformations. For the purposes of this study, ectasia/pyelectasis was defined as persistent dilatation of the renal pelvis documented on ultrasound and considered indicative of an underlying structural abnormality [21,22]. Complex anomalies were defined as the coexistence of two or more nephrourological malformations in the same patient, including combinations of hydronephrosis, ureteral dilation, vesicoureteral reflux, ureterocele, posterior urethral valves, or other congenital urinary tract abnormalities [23,24].

Abnormal studies without sufficient evidence of underlying anomalous urinary tract architecture were retained as abnormal non-malformative ultrasounds. This category included nonspecific or transient sonographic findings considered related to the acute infectious episode rather than to congenital structural abnormalities, such as focal parenchymal echogenicity changes, inflammatory renal alterations, mild pelvic fullness not meeting criteria for persistent pyelectasis or hydronephrosis, isolated bladder wall thickening, and other nonspecific abnormalities reported by the interpreting radiologist [19,20].

### 2.5. Exposures and Outcomes

The primary exposure of the study was the presence of nephrourological malformation (yes/no) in the entire cohort with interpretable ultrasound. As a secondary exposure, and only within the subgroup without final malformation, abnormal non-malformative ultrasound was compared against normal ultrasound. Outcomes of interest were: (a) documented outpatient follow-up after discharge; (b) referral to subspecialty care, mainly pediatric nephrology, pediatric urology, or other specialized care related to the case; and (c) subsequent hospital readmission documented in the institutional medical record. Variables reflecting greater clinical intensity during the index episode, such as prolonged hospital stay, were also explored. Hospital stay ≥7 days was evaluated as an exploratory indicator of prolonged hospitalization because this duration exceeds the typical length of inpatient management for uncomplicated pediatric UTI at our institution.

### 2.6. Clinical and Microbiological Variables

Potentially relevant demographic and clinical variables were recorded, including age, sex, birth weight, Capurro score (a neonatal assessment method used to estimate gestational age based on physical and neurological maturity criteria) [25,26], history of first UTI (defined as the first documented urinary tract infection episode recorded in the patient’s medical history at the time of the index hospitalization; patients with one or more previous documented UTI episodes were classified as having recurrent UTI), residence, and breastfeeding. In the microbiological component, the availability of urine culture, its final result as positive or negative, and the isolation of Escherichia coli or other microorganisms were recorded when the culture was interpretable. To avoid ambiguity in denominators, the main microbiological analyses were restricted to interpretable urine cultures, defined as those conclusively reported as positive or negative.

### 2.7. Statistical Analysis

In the entire cohort with interpretable renal ultrasound, primary comparisons were structured into two mutually exclusive contrasts: (1) malformation present versus absent; and (2) abnormal ultrasound without malformation versus normal ultrasound. Continuous variables were evaluated using an integrated strategy based on skewness and visual inspection of Q–Q plots. Absolute skewness values below 2 were considered broadly compatible with parametric assumptions when Q–Q plots did not show severe deviations from expected linearity. Under this criterion, age, birth weight, and Capurro score were analyzed using Welch’s *t*-test; length of hospital stay showed a skewed distribution and was summarized as median [interquartile range], with comparison by Mann–Whitney U test. All categorical variables were compared using Fisher’s exact test [27]. Analyses were performed using available-case analysis. No imputation procedures were applied, and the number of observations available for each variable is reported in the corresponding tables.

Crude associations between exposure and outcomes were expressed as relative risks (RR) with 95% confidence intervals. For the primary multivariable analysis, generalized linear models with Poisson distribution, log link, and HC3 robust standard errors were used to estimate adjusted risk ratios (aRR) for follow-up, referral, and readmission. Modified Poisson regression was selected because the outcomes of interest were relatively common, making odds ratios from logistic regression potentially less interpretable. This approach allows direct estimation of adjusted risk ratios while providing robust variance estimation given the number of events and covariates included in the models.

Models were adjusted for age in months, male sex, and history of first UTI, variables selected a priori based on clinical plausibility and consistent availability in the database. Given the limited sample size of some strata, analyses according to microbiological certainty were maintained as unadjusted (crude) bivariate comparisons and should be interpreted as exploratory sensitivity analyses. All P values were two-sided, and *p* < 0.05 was considered statistically significant. Analyses were performed using IBM SPSS Statistics, version 25 (IBM Corp., Armonk, NY, USA) [28].

### 2.8. Ethical Considerations

The protocol was approved by the Ethics and Research Committee of the General Hospital Zone No. 1 of IMSS Colima (Protocol R-2025-601-028, approval date: 24 April 2025). As this was a retrospective observational study based on medical records and without direct intervention on patients, the ethics committee waived informed consent according to applicable local regulations [29]. The study was written following the principles of the STROBE guideline for observational studies [30].

## 3. Results

**Figure 1:**
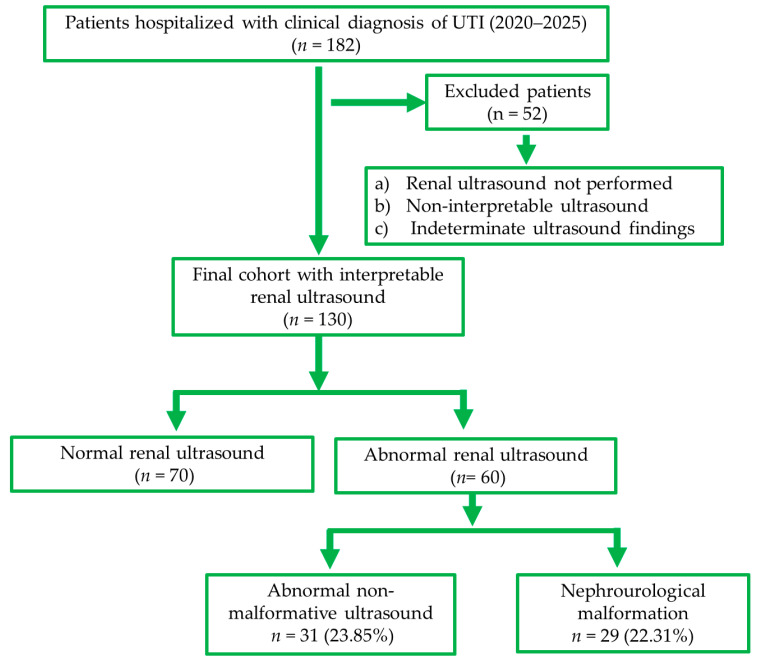
Flowchart of patient selection and ultrasonographic classification in the cohort.

A total of 182 patients were initially identified. After applying the predefined eligibility criteria, 130 children with interpretable renal ultrasound findings were included in the final analytical cohort. The process of patient selection and ultrasonographic classification is summarized in Figure 1.

As shown in Table 1, sex distribution differed significantly according to malformation status. Patients without malformation showed a clear female predominance, whereas the malformation group exhibited a more balanced sex distribution.

The spectrum of structural abnormalities within the malformation group was dominated by ectasia/pyelectasis, followed by complex anomalies and hydronephrosis. A small subset of patients presented pyeloureteral dilatation on the index ultrasound, which was subsequently confirmed as vesicoureteral reflux by voiding cystourethrography during follow-up (Table 2). The coexistence of more than one structural alteration in several patients supports the use of the term malformative phenotype rather than the notion of isolated findings. In contrast, non-malformative ultrasonographic abnormalities were predominantly grouped around inflammatory or scar-related changes associated with the index episode.

The comparison between the subgroups with and without malformation confirmed that the structural malformation phenotype was associated with a greater clinical burden during and after the index episode. Mean age was higher in the malformation group, although without statistical significance (see Table 3). Birth weight and Capurro score were comparable between groups. Length of hospital stay was significantly longer in the presence of malformation (median 3.00 vs. 5.00 days; *p* = 0.015) (Table 3).

The largest absolute differences between groups were observed for outpatient follow-up, referral to subspecialty care, and hospital readmission, all of which occurred more frequently among patients with nephrourological malformations than among those without malformations.

Regarding outcomes, the differences were consistent and clinically relevant. Outpatient follow-up occurred in 79.31% of patients with malformation compared with 44.55% of those without malformation (*p* = 0.001). Referral to subspecialty care was documented in 79.31% versus 49.50% (*p* = 0.005), and hospital readmission in 44.83% versus 13.86%, respectively (*p* = 0.001) (see Table 4). The absolute difference in outpatient follow-up between groups was 34.8 percentage points, whereas the absolute differences for referral to subspecialty care and hospital readmission were 29.8 and 31.0 percentage points, respectively. These findings were further reflected in the association analyses (Table 4). Nephrourological malformation was associated with a higher probability of outpatient follow-up (RR 1.78, 95% CI 1.34–2.37), a greater likelihood of referral to subspecialty care (RR 1.60, 95% CI 1.22–2.10), and an increased risk of hospital readmission (RR 3.23, 95% CI 1.72–6.08).

The differences remained consistent in the multivariable analysis, in which nephrourological malformation maintained an independent association with greater clinical burden following the index episode (Table 5). After adjustment for age in months, sex, and first UTI, the presence of malformation was associated with a higher probability of outpatient follow-up (aRR 1.72; 95% CI 1.25–2.37; *p* = 0.001), a greater likelihood of referral to subspecialty care (aRR 1.59; 95% CI 1.17–2.16; *p* = 0.003), and an increased risk of hospital readmission (aRR 3.38; 95% CI 1.58–7.23; *p* = 0.002).

In contrast, within the subgroup without malformation, the presence of an abnormal non-malformative ultrasound did not show adjusted associations with outpatient follow-up (aRR 1.06; 95% CI 0.63–1.76; *p* = 0.834), referral to subspecialty care (aRR 1.18; 95% CI 0.76–1.85; *p* = 0.465), or hospital readmission (aRR 2.27; 95% CI 0.69–7.46; *p* = 0.175). Taken together, these results indicate that the principal prognostic factor is the structural malformation phenotype rather than non-malformative ultrasonographic abnormalities.

### Analysis According to Microbiological Diagnostic Certainty

Forty-seven of 130 patients (36.15%) had microbiologically confirmed UTI, whereas 83/130 (63.85%) remained within the category of probable clinical/microbiologically unconfirmed UTI. Malformation was more frequent in the microbiologically confirmed subcohort than in the non-confirmed group (34.04% vs. 15.66%; *p* = 0.027), suggesting that the structural phenotype is associated not only with greater subsequent clinical burden, but also with a higher probability of bacteriological documentation (Table 6).

A second analysis was performed in a subsample restricted to patients with interpretable urine cultures, defined exclusively as either positive or negative cultures (Table 7). Within this stratum, patients with malformation maintained a higher rate of urine culture positivity (66.67% vs. 38.75%; *p* = 0.020) and a higher frequency of *Escherichia coli* isolation when the denominator included all interpretable cultures (54.17% vs. 30.00%; *p* = 0.050). However, when the denominator was restricted only to positive cultures, *Escherichia coli* represented similar proportions of isolates in both groups (81.25% vs. 77.42%; *p* = 1.000).

This distinction is important because it suggests that the principal difference between groups is not bacterial ecology, but rather a higher probability of microbiological confirmation among patients with malformation.

Stratification according to microbiological diagnostic certainty reinforced the findings observed in the overall cohort. Among the 47 cases with microbiologically confirmed UTI, the presence of malformation was associated with greater outpatient follow-up (81.25% vs. 35.48%; RR 2.29, 95% CI 1.34–3.91; *p* = 0.005), greater referral to subspecialty care (81.25% vs. 38.71%; RR 2.10, 95% CI 1.25–3.53; *p* = 0.007), and higher hospital readmission (37.50% vs. 6.45%; RR 5.81, 95% CI 1.31–25.68; *p* = 0.013) (Table 8).

Within the probable clinical/microbiologically unconfirmed UTI stratum, the direction of the effect remained consistent. Outpatient follow-up was significantly more frequent among patients with malformation than among those without malformation (76.92% vs. 48.57%; RR 1.58, 95% CI 1.08–2.32; *p* = 0.043). Referral to subspecialty care showed a similar direction of effect (RR 1.42, 95% CI 0.98–2.05; *p* = 0.198), and hospital readmission remained more frequent among patients with malformation (RR 3.14, 95% CI 1.46–6.77; *p* = 0.008) (Table 8).

The direction of the association was consistent across both diagnostic certainty strata. Notably, outpatient follow-up and hospital readmission remained significantly associated with malformation even within the probable clinical/microbiologically unconfirmed UTI subgroup, although estimates in this stratum should still be interpreted cautiously because of the limited sample size.

## 4. Discussion

Within a real-world hospital cohort of children with a clinical diagnosis of UTI and interpretable renal ultrasound, the greater subsequent clinical burden was not concentrated in the broad and nonspecific category of “abnormal ultrasound,” but rather in the nephrourological malformation phenotype. When ultrasonographic abnormalities are grouped together as a single category, the finding becomes heterogeneous and clinically ambiguous. In contrast, by separating structural malformations from non-malformative alterations, a consistent result emerged: malformations were associated with longer hospital stays, higher frequency of outpatient follow-up, greater referral to subspecialty care, and increased risk of hospital readmission, whereas non-malformative abnormalities did not reproduce this pattern in adjusted analyses. These findings should not be interpreted as indicating that non-malformative ultrasounds were normal, but rather that they represented abnormalities without evidence of an underlying congenital nephrourological malformation. This distinction is clinically relevant because it suggests that the prognostic value of renal ultrasound after pediatric UTI may depend more on the identification of structural anomalies than on the mere presence of nonspecific ultrasonographic abnormalities.

This finding does not contradict, but rather refines, the available literature regarding the clinical utility of renal ultrasound after pediatric UTI. In the systematic review and meta-analysis by Yang and colleagues [10], approximately one in every four to five children presented some abnormality on ultrasound after a first febrile UTI, but only around one in 32 had an abnormality considered clinically important, that is, with the potential to modify clinical management. That same study also highlighted the considerable heterogeneity between studies and the scarcity of robust longitudinal outcomes, especially regarding subsequent healthcare utilization and clinical evolution [10].

In our cohort, the frequency of globally abnormal ultrasound was higher (46.15%), which is expected considering that this was a hospital-based population, not restricted to a first outpatient febrile UTI, and enriched with patients of greater clinical complexity. However, the original contribution of the present study does not lie in showing a higher proportion of abnormal studies, but rather in demonstrating that not all ultrasonographic abnormalities carry the same clinical weight. In particular, the excess subsequent clinical burden was concentrated in the subgroup with adjudicated structural malformations, whereas non-malformative, inflammatory, or nonspecific ultrasonographic alterations did not reproduce this pattern in adjusted analyses [10,31].

A Finnish study [32] provides a useful framework for interpreting our results, although it is not strictly superimposable to our cohort. In a population of 2050 children aged 0 to 16 years undergoing urinary ultrasound after UTI, the authors found abnormalities in 5.7% of cases and observed a higher probability of abnormal findings in males, in non-E. coli UTI, and in recurrent UTI. Furthermore, they showed that an age-restricted imaging strategy could miss a relevant fraction of abnormalities, particularly in the subgroup with pyelonephritis. However, that study excluded patients with previously known urinary pathology and did not classify certain findings attributable to acute infection as abnormalities; therefore, its prevalence estimate represents a more restricted definition of the abnormal ultrasonographic phenotype [32]. From this perspective, the greater burden of abnormality observed in our cohort should not be interpreted solely as a quantitative difference, but rather as the expression of a different clinical scenario: A retrospective single-center hospital-based cohort with greater complexity and explicit adjudication of the structural component. Thus, the added value of our study lies not only in detecting more abnormalities, but in demonstrating that the excess subsequent clinical burden is concentrated in the subgroup with structural malformations, whereas non-malformative alterations do not reproduce this pattern of follow-up, referral, and readmission [32].

Congenital anomalies of the kidney and urinary tract (CAKUT) constitute the most frequent group of malformations associated with kidney disease in childhood and represent the leading cause of kidney failure in children worldwide. Their importance does not depend solely on the initial anatomical finding, but on their capacity to produce urinary stasis, obstruction, reflux, recurrent infections, and the need for longitudinal nephrological or urological surveillance [33,34]. In this context, the fact that the malformation group presented more follow-up, more referrals, and more readmissions goes beyond a mere statistical association; rather, it reflects the expected clinical manifestation of a structural phenotype requiring greater medical care. Therefore, identifying the subgroup with relevant downstream consequences in the patient trajectory is of particular importance [35,36].

An important contribution of the present manuscript is that it clearly addresses a recurring issue in real-world clinical practice across different regions: not all hospitalized patients treated as UTI have the same degree of microbiological confirmation. Guideline literature continues to consider an appropriately obtained urine culture as the central element for diagnostic confirmation, and current reviews maintain that the diagnosis of UTI should rely on a combination of compatible clinical presentation, urinary inflammation, and positive culture. [37,38] However, real-world cohorts, especially hospital-based cohorts, inevitably include patients with negative, pending, unavailable, or inconclusive cultures who were nevertheless clinically managed as UTI. Instead of obscuring this problem or labeling the entire cohort as microbiologically confirmed, the present study adopted a strategy aligned with real-world hospital practice: working with a primary cohort of clinical UTI and a subcohort of microbiologically confirmed UTI. This design does not weaken the manuscript; on the contrary, it strengthens it, because it demonstrates that the prognostic role of malformation does not disappear after stratification by diagnostic certainty and, within the confirmed subcohort, even becomes more pronounced. This persistence across strata reinforces the robustness of the principal finding and increases its clinical credibility [36].

Sex distribution also provided interesting findings. The cohort without malformation showed a clear female predominance, whereas the malformation group exhibited a nearly balanced sex distribution [39,40]. This pattern is consistent with more recent post-UTI imaging cohorts that have found a greater probability of abnormal ultrasonographic findings in males. In other words, although pediatric UTI overall remains more frequent in girls, the presence of a structural phenotype appears to attenuate this female predominance and shift group composition toward greater male representation [41]. This suggests that sex not only modulates the risk of UTI, but also the probability that the episode reflects an underlying pathological urinary tract architecture.

From a microbiological perspective, the malformation group showed greater urine culture positivity when analyses were restricted to interpretable studies. Nevertheless, within the stratum of children with positive cultures, Escherichia coli remained the predominant microorganism in similar proportions between groups, suggesting that the principal difference does not lie in bacterial ecology, but rather in the greater probability of microbiologically documenting infection in the presence of malformation [42,43]. This finding is important because it suggests that the principal microbiological difference is not the replacement of one etiological ecosystem by another completely different one, but rather a greater ease in microbiologically documenting infection in the presence of a predisposing structural substrate. From a pathophysiological perspective, this is consistent with the notion that malformation favors bacterial persistence, inefficient drainage, or clinical recurrence [44,45], rather than a radically different bacterial ecology.

The clinical implications of the study are direct. Recent guidelines for complicated pediatric UTI recommend follow-up and serial imaging in children with significant anatomical or functional anomalies and in those with recurrent infections. Our results are fully consistent with this rationale: the malformation group was precisely the one concentrating the highest density of follow-up, specialized referral, and readmission. Therefore, the manuscript may argue that uro-renal ultrasound should not be understood solely as an anatomical screening test, but also as an initial tool for stratifying future healthcare burden in hospital settings of clinical UTI [46,47]. From a healthcare organization perspective, this idea is relevant: not all children with ultrasonographic findings require the same level of follow-up, but there is an identifiable subgroup from the index episode onward that will probably benefit from a more structured care pathway, with closer nephrological/urological surveillance and reduced fragmentation of care.

The study has several strengths. First, it works with a real-world clinical cohort, which improves external relevance for hospital settings where microbiological certainty is not always uniform. Second, it avoids a frequent error in retrospective literature: treating every ultrasonographic abnormality as if it had the same clinical meaning. Final adjudication of malformation status allowed a conceptually cleaner separation between structural findings and non-malformative alterations. Third, the study does not merely describe imaging prevalences but links these findings to tangible subsequent clinical outcomes: follow-up, referral, and readmission. Fourth, the consistency of the pattern across different analyses (crude, adjusted, and stratified by microbiological certainty) increases the internal robustness of the results.

The principal limitation of the study remains its retrospective nature. Another limitation is the relatively small sample size after stratification, especially within the microbiologically confirmed infection subcohort, which widened some confidence intervals. Nevertheless, the direction of the effect remained stable, particularly for readmission, supporting the robustness of the central finding. The study cohort was also restricted to patients with interpretable renal ultrasound examinations, which may have introduced selection bias by excluding children without available or classifiable imaging. Although a structured adjudication process was applied, some degree of misclassification of ultrasonographic findings cannot be completely excluded, particularly among abnormalities classified as non-malformative. In addition, residual confounding remains possible because certain factors influencing clinical management and outcomes could not be fully captured from retrospective medical records. Another potential limitation is diagnostic ascertainment bias. Although urine culture is routinely requested in hospitalized children with suspected UTI at our institution, clinicians may have had a lower threshold for pursuing microbiological confirmation or repeating diagnostic evaluations in patients with known or suspected nephrourological abnormalities. Consequently, the higher frequency of microbiologically confirmed UTI observed in the malformation group may partly reflect increased diagnostic scrutiny in addition to biological susceptibility. This possibility cannot be excluded in a retrospective study and should be considered when interpreting microbiological comparisons. Finally, the study was conducted at a single referral center, which may limit the generalizability of the findings to other healthcare settings. Therefore, the present results should be interpreted as observational associations rather than causal relationships and should be confirmed in larger multicenter and prospective studies.

## 5. Conclusions

In this real-world hospital cohort of children with presumed urinary tract infection and interpretable renal ultrasound, the subsequent clinical burden was concentrated in the nephrourological malformation phenotype rather than in the broad category of “abnormal ultrasound.” Structural malformations were consistently associated with longer hospital stay, greater outpatient follow-up, increased referral to subspecialty care, and higher risk of hospital readmission, whereas non-malformative ultrasonographic abnormalities did not reproduce this pattern after adjustment.

These findings suggest that the clinical value of renal ultrasound after pediatric UTI lies not merely in the indiscriminate detection of abnormalities, but in its ability to identify a subgroup of patients with underlying structural vulnerability and greater future healthcare needs. The persistence of these associations across analyses stratified by microbiological diagnostic certainty further reinforces the robustness and clinical relevance of the malformative phenotype.

From a practical perspective, renal ultrasound may serve not only as an anatomical imaging tool, but also as an early prognostic stratification instrument capable of identifying children who may benefit from closer nephrological or urological surveillance and more structured longitudinal care pathways.

## Figures and Tables

**Table 1:** Sex distribution according to final malformation status.

Sex	Without Malformation (n = 101)	With Malformation (n = 29)	*p* Between Subgroups
Male	24 (23.76)	14 (48.28)	0.019
Female	77 (76.24)	15 (51.72)	
P within subgroup	<0.001	1.000	

Data are presented as *n* (% by column). The *p* value between subgroups was obtained using Fisher’s exact test and compares sex composition between patients with and without malformation. The *p* value within subgroup was obtained using Fisher’s exact test.

**Table 2:** Spectrum of structural abnormalities within the final nephrourological malformation group.

Structural/Phenotypic Alteration	Frequency
Ectasia/Pyelectasis	19/29 (65.52%)
Complex anomaly	9/29 (31.03%)
Hydronephrosis	8/29 (27.59%)
Ureterocele	3/29 (10.34%)
Pyeloureteral dilatation *	3/29 (10.34%)
Posterior urethral valves	2/29 (6.90%)
Lithiasis/Nephrocalcinosis	1/29 (3.45%)
Concomitant inflammatory/scarring changes	1/29 (3.45%)

Alterations were not mutually exclusive; the same patient could contribute to more than one category. Percentages were calculated using the final malformation cohort as the denominator (*n* = 29). * Pyeloureteral dilatation detected on the index renal ultrasound. These cases were subsequently confirmed as vesicoureteral reflux by voiding cystourethrography during follow-up.

**Table 3:** Clinical characteristics and outcomes according to final malformation status in 130 patients with interpretable renal ultrasound.

Variable	Available (*n* *)	Overall	Without Malformation	With Malformation	*p* Value	Statistical Test
Age, months	130	53.66 ± 54.47	49.64 ± 52.44	67.66 ± 59.91	0.150	Welch’s *t*-test
Birth weight, kg	101	3.22 ± 0.53	3.19 ± 0.52	3.31 ± 0.58	0.441	Welch’s *t*-test
Capurro score, weeks	115	38.53 ± 1.66	38.53 ± 1.66	38.54 ± 1.69	0.971	Welch’s *t*-test
Length of hospital stay, days	128	4.00 [2.00–6.00]	3.00 [2.00–5.00]	5.00 [3.00–7.00]	0.015	Mann–Whitney U test
Male sex	130	38/130 (29.23%)	24/101 (23.76%)	14/29 (48.28%)	0.019	Fisher’s exact test
Age < 12 months	130	38/130 (29.23%)	29/101 (28.71%)	9/29 (31.03%)	0.820	Fisher’s exact test
Breastfeeding	127	93/127 (73.23%)	70/100 (70.00%)	23/27 (85.19%)	0.144	Fisher’s exact test
First UTI	127	92/127 (72.44%)	76/99 (76.77%)	16/28 (57.14%)	0.055	Fisher’s exact test
Residence in Colima	129	113/129 (87.60%)	87/100 (87.00%)	26/29 (89.66%)	1.000	Fisher’s exact test
Outpatient follow-up	130	68/130 (52.31%)	45/101 (44.55%)	23/29 (79.31%)	0.001	Fisher’s exact test
Referral to subspecialty care	130	73/130 (56.15%)	50/101 (49.50%)	23/29 (79.31%)	0.005	Fisher’s exact test
Hospital readmission	130	27/130 (20.77%)	14/101 (13.86%)	13/29 (44.83%)	0.001	Fisher’s exact test
Hospital stay ≥ 7 days	128	26/128 (20.31%)	16/99 (16.16%)	10/29 (34.48%)	0.039	Fisher’s exact test

Continuous variables are presented as mean ± standard deviation when analyzed using parametric methods and as median [interquartile range] when analyzed using non-parametric methods. Categorical variables are presented as n/N (%). *p* values were obtained using Welch’s *t*-test, Mann–Whitney U test, or Fisher’s exact test, as appropriate. The available number of observations for each variable is indicated and corresponds to the denominator used in the analyses. * The number of patients analyzed for each variable is indicated. First UTI refers to patients whose index hospitalization corresponded to their first documented urinary tract infection episode.

**Table 4:** Crude associations between nephrourological malformation and subsequent clinical outcomes.

Exposure	Outcome	*n*	Exposed	Non-Exposed	Crude RR (95% CI)	*p* Value
Malformation yes vs. no	Outpatient follow-up	130	23/29 (79.31%)	45/101 (44.55%)	1.78 (1.34–2.37)	0.001
Malformation yes vs. no	Referral to subspecialty care	130	23/29 (79.31%)	50/101 (49.50%)	1.60 (1.22–2.10)	0.005
Malformation yes vs. no	Hospital readmission	130	13/29 (44.83%)	14/101 (13.86%)	3.23 (1.72–6.08)	0.001
Abnormal ultrasound without malformation vs. normal ultrasound	Outpatient follow-up	101	14/31 (45.16%)	31/70 (44.29%)	1.02 (0.64–1.63)	1.000
Abnormal ultrasound without malformation vs. normal ultrasound	Referral to subspecialty care	101	17/31 (54.84%)	33/70 (47.14%)	1.16 (0.78–1.74)	0.522
Abnormal ultrasound without malformation vs. normal ultrasound	Hospital readmission	101	6/31 (19.35%)	8/70 (11.43%)	1.69 (0.64–4.47)	0.352

Absolute risks in exposed and non-exposed groups, crude risk ratios (RR) with 95% confidence intervals, and *p* values obtained using Fisher’s exact test are presented. For comparisons involving malformation status, the exposed group corresponds to patients with nephrourological malformations and the non-exposed group to patients without malformations. For comparisons involving abnormal non-malformative ultrasound findings, the exposed group corresponds to patients with abnormal non-malformative ultrasound findings and the non-exposed group to patients with normal ultrasound findings.

**Table 5:** Adjusted models (adjusted for age in months, male sex, and first UTI) for subsequent clinical outcomes.

Comparison	Outcome	*n* *	aRR	95% CI	*p* Value
Malformation yes vs. no	Outpatient follow-up	127	1.72	1.25–2.37	0.001
Malformation yes vs. no	Referral to subspecialty care	127	1.59	1.17–2.16	0.003
Malformation yes vs. no	Hospital readmission	127	3.38	1.58–7.23	0.002
Abnormal ultrasound without malformation vs. normal ultrasound	Outpatient follow-up	99	1.06	0.63–1.76	0.834
Abnormal ultrasound without malformation vs. normal ultrasound	Referral to subspecialty care	99	1.18	0.76–1.85	0.465
Abnormal ultrasound without malformation vs. normal ultrasound	Hospital readmission	99	2.27	0.69–7.46	0.175

Adjusted risk ratios (aRR) with 95% confidence intervals are presented. Models were estimated using modified Poisson regression with log link and HC3 robust standard errors, adjusted for age in months, male sex, and first UTI. The comparison between abnormal ultrasound without malformation and normal ultrasound was restricted to the subgroup without final malformation. * Although the total sample consisted of 130 patients, complete data were not available for all variables (including adjustment variables); therefore, the number of patients with complete data used for each analysis is indicated.

**Table 6:** Diagnostic certainty framework of the analytical cohort with interpretable renal ultrasound.

Diagnostic Category	*n*	With Malformation	Without Malformation	*p* Value
Microbiologically confirmed UTI	47	16/47 (34.04%)	31/47 (65.96%)	0.027
Probable clinical/microbiologically unconfirmed UTI	83	13/83 (15.66%)	70/83 (84.34%)	

The primary cohort was defined as hospitalized presumed UTI with interpretable renal ultrasound. Microbiologically confirmed UTI corresponded to a positive urine culture for a recognized uropathogen. The category of probable clinical/microbiologically unconfirmed UTI included negative, unavailable, missing, or inconclusive cultures within a cohort clinically treated as UTI. The P value compares the prevalence of malformation between diagnostic certainty strata using Fisher’s exact test.

**Table 7:** Microbiology restricted to interpretable urine cultures according to final malformation status.

Variable	Without Malformation	With Malformation	*p* Value
Interpretable urine cultures, n/N within subgroup	80/101 (79.21%)	24/29 (82.76%)	0.793
Positive urine culture, n/N interpretable	31/80 (38.75%)	16/24 (66.67%)	0.020
Negative urine culture, n/N interpretable	49/80 (61.25%)	8/24 (33.33%)	0.020
Escherichia coli, n/N interpretable	24/80 (30.00%)	13/24 (54.17%)	0.050
Escherichia coli among positive cultures, n/N positive	24/31 (77.42%)	13/16 (81.25%)	1.000
Other microorganism among positive cultures, n/N positive	7/31 (22.58%)	3/16 (18.75%)	1.000

Percentages of positivity and negativity were calculated using only patients with interpretable urine cultures (positive or negative) as the denominator. Percentages of *Escherichia coli* are expressed both over the total number of interpretable cultures and, in a secondary analysis, over the subset of positive cultures. *p* values were obtained using Fisher’s exact test.

**Table 8:** Association analysis between the presence of nephrourological malformation and subsequent follow-up, specialist referral, or hospital readmission stratified by microbiological diagnostic certainty.

Stratum	Outcome	With Malformation	Without Malformation	RR (95% CI)	*p* Value
Microbiologically confirmed UTI	Outpatient follow-up	13/16 (81.25%)	11/31 (35.48%)	2.29 (1.34–3.91)	0.005
Microbiologically confirmed UTI	Referral to subspecialty care	13/16 (81.25%)	12/31 (38.71%)	2.10 (1.25–3.53)	0.007
Microbiologically confirmed UTI	Hospital readmission	6/16 (37.50%)	2/31 (6.45%)	5.81 (1.31–25.68)	0.013
Probable clinical/microbiologically unconfirmed UTI	Outpatient follow-up	10/13 (76.92%)	34/70 (48.57%)	1.58 (1.08–2.32)	0.043
Probable clinical/microbiologically unconfirmed UTI	Referral to subspecialty care	10/13 (76.92%)	38/70 (54.29%)	1.42 (0.98–2.05)	0.198
Probable clinical/microbiologically unconfirmed UTI	Hospital readmission	7/13 (53.85%)	12/70 (17.14%)	3.14 (1.46–6.77)	0.008

Each stratum summarizes bivariate comparisons between patients with and without malformation within different levels of diagnostic certainty. Given the limited sample size, these analyses should be interpreted as descriptive sensitivity analyses rather than conclusive evidence of interaction.

## Data Availability

The data supporting the findings of this study are not publicly available due to privacy and ethical restrictions related to patient confidentiality. De-identified data may be available from the corresponding author upon reasonable request and with permission from the Ethics and Research Committee of the General Hospital Zone No. 1 of IMSS-Colima.
